# Innovations in Proteomic Technologies and Artificial Neural Networks: Unlocking Milk Origin Identification

**DOI:** 10.3390/biotech14020033

**Published:** 2025-04-28

**Authors:** Achilleas Karamoutsios, Emmanouil D. Oikonomou, Chrysoula (Chrysa) Voidarou, Lampros Hatzizisis, Konstantina Fotou, Konstantina Nikolaou, Evangelia Gouva, Evangelia Gkiza, Nikolaos Giannakeas, Ioannis Skoufos, Athina Tzora

**Affiliations:** 1 Laboratory of Animal Health, Hygiene and Food Quality, School of Agriculture, University of Ioannina, 47100 Arta, Greece; a.karamoutsios@uoi.gr (A.K.); xvoidarou@uoi.gr (C.V.); kfotou@uoi.gr (K.F.); knikolaou@uoi.gr (K.N.); egouva@uoi.gr (E.G.); 2 Human Computer Interaction Laboratory, Department of Informatics and Telecommunications, University of Ioannina, 47100 Arta, Greece; e.oikonomou@uoi.gr (E.D.O.); giannakeas@uoi.gr (N.G.); 3 Laboratory of Animal Science, Nutrition and Biotechnology, School of Agriculture, University of Ioannina, 47100 Arta, Greece; lamprosxatz@uoi.gr (L.H.); jskoufos@uoi.gr (I.S.); 4 P.G. Nikas SA, Department of Regulatory Affairs & Quality Assurance, Agios Stefanos, 14565 Attica, Greece; egkiza@nikas.gr

**Keywords:** ruminant milk, proteomics, mass spectrometry, MALDI-TOF MS, machine learning, neural networks, food quality assurances

## Abstract

Milk’s biological origin determination, including its adulteration and authenticity, presents serious limitations, highlighting the need for innovative advanced solutions. The utilisation of proteomic technologies combined with personalised algorithms creates great potential for a more comprehensive approach to analysing milk samples effectively. The current study presents an innovative approach utilising proteomics and neural networks to classify and distinguish bovine, ovine and caprine milk samples by employing advanced machine learning techniques; we developed a precise and reliable model capable of distinguishing the unique mass spectral signatures associated with each species. Our dataset includes a diverse range of mass spectra collected from milk samples after MALDI-TOF MS (Matrix-assisted laser desorption/ionization-time of flight mass spectrometry) analysis, which were used to train, validate, and test the neural network model. The results indicate a high level of accuracy in species identification, underscoring the model’s potential applications in dairy product authentication, quality assurance, and food safety. The current research offers a significant contribution to agricultural science, providing a cutting-edge method for species-specific classification through mass spectrometry. The dataset comprises 648, 1554, and 2392 spectra, represented by 16,018, 38,394, and 55,055 eight-dimensional vectors from bovine, caprine, and ovine milk, respectively.

## 1. Introduction

Dairy farming constitutes a fundamental component of global agriculture. It involves the production of milk from a diverse array of domesticated mammals—not only from cows, goats, and ewes but also from buffalos, camels, donkeys, and other species. This sector is critical to food production, supplying dairy products that are staple components of diets around the world [1]. These products deliver essential nutrients, including calcium, proteins, and vitamins, which are vital for human health. Beyond its nutritional contributions, the dairy industry plays a crucial socioeconomic role. It provides financial support to millions of farmers and farmworkers, especially in rural areas where dairy farming frequently represents a primary source of income. Moreover, the sector stimulates economic stability and growth by creating employment opportunities and generating demand for related agricultural inputs such as feed production, veterinary services, and dairy processing. However, the industry faces significant challenges regarding milk quality and safety. A major concern is milk adulteration—the practice of incorporating cheaper or non-milk substances into milk. This not only deceives consumers but also raises serious public health issues, as adulterated milk may harbour pathogens, antibiotics, or other harmful compounds. Ensuring the purity and safety of milk is therefore essential for protecting consumer health and sustaining the economic benefits of dairy farming. Accurate labelling of food products, particularly regarding their value-determining ingredients, is not only a regulatory mandate but also a critical factor in fostering consumer trust and empowerment [2,3]. A prevalent challenge in the dairy industry is the practice of incorporating relatively low-value milk into premium sheep’s, goat’s, or buffalo’s milk designated for cheese and other products. This form of adulteration has raised significant concerns among consumer protection laboratories. Such malpractice adversely affects consumers financially and poses health risks, especially for those with sensitivities and allergies [4]. These issues underscore the urgent need for enhanced oversight mechanisms and stricter regulatory frameworks to ensure transparency and accountability in the marketplace.

The detailed analysis of key whey and casein proteins has proven essential for identifying the animal species origin of milk, as each type of mammalian milk exhibits a distinct proteomic pattern. This unique pattern can be examined using a variety of analytical strategies [5]. Techniques such as capillary electrophoresis (CE) and liquid chromatography (LC) paired with detectors like ultraviolet absorption (UV) or mass spectrometry (MS) have been recognized for their precision in differentiating milk proteins across species [6,7,8,9,10]. However, despite their accuracy, these methods are often cumbersome due to their demanding operational requirements.

In response to these challenges, the advent of matrix-assisted laser desorption/ionization time-of-flight mass spectrometry (MALDI-TOF MS) represents a pivotal breakthrough in food analysis. This cutting-edge technology offers a highly reliable and efficient method for accurately identifying the specific animal species of milk used in the production of cheese and a broad range of other dairy products. By significantly enhancing the precision of food labelling, MALDI-TOF MS plays a crucial role in protecting consumer health and bolstering market trust [11,12,13,14].

Protein and peptide fingerprints can be analysed using matrix-assisted laser desorption/ionization time-of-flight mass spectrometry (MALDI-TOF MS), a technique that has become a cornerstone in food analysis laboratories for the identification of microorganisms [15,16,17,18,19,20]. Furthermore, MALDI-TOF MS has demonstrated its effectiveness in determining and discriminating among various animal species in muscle meat, scallops, shrimp, fish, and edible insects [21,22,23,24,25]. In the dairy products sector, this technique has been employed to distinguish between cow’s and she-donkey’s milk as well as between goat’s and sheep’s milk. Additionally, it has proven useful for differentiating buffalo mozzarella or ricotta from cow’s milk cheese following tryptic digestion [26,27,28,29,30,31,32].

Despite the well-recognized potential of MALDI-TOF-MS for detecting low-value milk adulteration in high-value milk at the intact protein level, only a limited number of studies have progressed beyond qualitative analysis to assess its quantitative effectiveness in measuring milk adulteration [33,34,35].

The integration of neural networks into the analysis and classification of MALDI-TOF MS proteomic profiles offers substantial advantages. Mass spectrometry delivers high-resolution, comprehensive data on the molecular composition of milk, while neural networks excel at uncovering intricate patterns within this data. This combination enables the precise identification of species-specific mass spectral signatures, facilitating the highly accurate detection of impurities, the evaluation of nutritional value, and even the determination of the milk product’s origin. Throughout this iterative process, the neural networks autonomously optimize their parameters to refine decision making, enhancing performance without direct human intervention [36,37,38].

A recent systematic review revealed that only 46 studies have applied machine learning methodologies to diverse facets of dairy science—including quality control, manufacturing processes, functionality, authentication, composition analysis, sensory evaluation, and adulteration detection [39]. Remarkably, no study to date has harnessed MALDI-TOF mass spectrometry data on milk casein proteins in tandem with a Feedforward Neural Network (FNN) for spectral interpretation aimed at milk classification. This gap underscores a compelling frontier for dairy research, where the fusion of advanced proteomic techniques and sophisticated machine learning holds the promise of revolutionizing milk authentication and quality assurance.

In the current study, neural networks were applied to classify bovine, ovine, and caprine milk samples based on their mass spectrometry profiles corresponding to casein proteins [14,37,40]. The dataset comprised a diverse range of mass spectra from milk samples, which were systematically divided into training, validation, and testing sets for model development. This approach provides a detailed comparison of proteomic profiles across different milk types and offers a scientifically robust method for dairy product authentication. The findings contribute to a more objective assessment of product authenticity and quality, supporting ongoing efforts in quality control and regulatory compliance.

## 2. Materials and Methods

**Figure 1:**
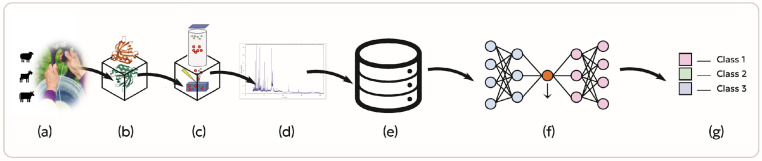
(**a**) Milk sampling, (**b**) casein extraction, (**c**) MALDI-TOF analysis, (**d**) spectra database, (**e**) data extraction, (**f**) neural network training, (**g**) animal species discrimination.

The current study is divided into four distinct stages—sample collection and preparation, mass spectrometry analysis, data extraction, and neural network model development—with each stage meticulously executed to ensure accuracy, reliability, and reproducibility (Figure 1). In particular, the mass spectrometry analysis stage employs MALDI-TOF MS, which offers significant advantages over LC–MS and GC–MS by reducing sample preparation requirements and eliminating the need for complex chromatography and costly consumables. This streamlined workflow facilitates high-throughput, cost-effective, and reliable analysis for routine food authentication and quality control applications [11,32].

### 2.1. Sample Collection and Preparation

This protocol was optimised and implemented following an extensive review of the relevant literature [14,41], with minor adjustments. The work was performed at the Laboratory of Animal Health, Hygiene, and Food Quality at the University of Ioannina, where the required analyses were completed. The process consists of three distinct phases, as outlined below:

#### 2.1.1. Milk Sampling and Laboratory Acquisition

**Figure 2:**
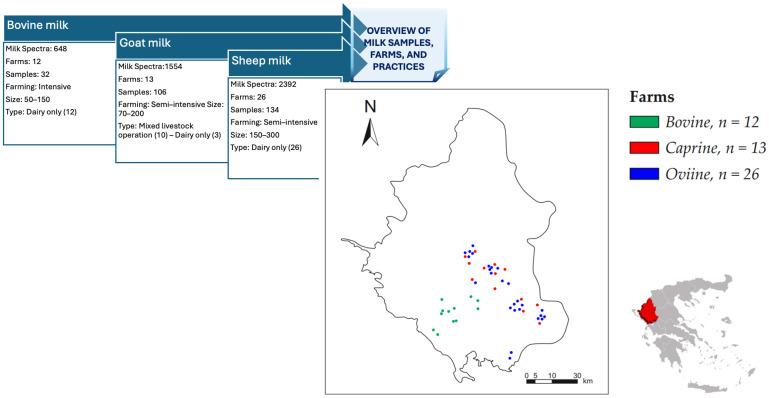
Schematic Overview of Milk Samples and Farm Characteristics by Species, from the Epirus region of Greece.

Dairy farms from the Epirus region of Greece were systematically selected for milk sampling conducted between November 2023 and May 2024.The small ruminant farms (250–400 dairy animals per farm) were classified as semi-intensive based on EFSA criteria—considering factors such as human contact, concentrate administration, pasture access, and pasture management [42]. The dairy cow housing, with 50–215 animals each, was characterized as intensive. Farms were randomly selected from a comprehensive regional list, and those with suboptimal bulk tank records (e.g., elevated bacterial counts or unsatisfactory somatic cell counts) were excluded to ensure sample integrity [43]. In each farm, veterinary professionals conducted the sampling after recording individual animal identities and performing clinical examinations to exclude animals with mastitis or other diseases. A total of 272 milk samples were analysed, and detailed information regarding sample sizes for each species, farm location, herd size, and production system is provided in Figure 2.

Mammary secretions were collected in sterile, labelled containers and promptly transported under controlled storage conditions (2–8 °C) to the laboratory for further analysis [19].

#### 2.1.2. Protein/Peptide Precipitation

The extraction protocol was specifically designed to enrich casein proteins (using acetic acid precipitation), ensuring that the subsequent MALDI-TOF MS spectra predominantly reflect casein-derived peptides and proteins. For each milk sample, after reaching room temperature, a 30 mL amount was transferred to a 50 mL Falcon-type container and centrifuged at 3345× *g* rpm at 8 °C for 12 min in a refrigerated centrifuge (Hettich, Rotina 35R, Kirchlengern, Germany). The samples were transferred to the bench, and using a spatula, the fat layer was removed from the walls of the tube and discarded. This was followed by filtration to eliminate any remaining fat. After this, 3–4 drops of 50% acetic acid (Fluka, Waltham, MA, USA) were added to the skimmed milk, and the mixture was heated in a water bath at a temperature of 40–45 °C for at least 15 min, until a sufficient amount of casein had precipitated. At this stage, large clots were typically visible within the skimmed milk. The liquid containing the sediment was then transferred to 1.5 mL tubes. After thorough mixing and homogenization, the tubes were centrifuged at 12,000–14,000× *g* rcf in a bench centrifuge for 2 min. Approximately 1 mL of ultrapure water (UltraPure, Distilled Water, Invitrogen, Carlsbad, CA, USA) was added to the mixture and stirred with a spatula. The mixture was then centrifuged again at 12,000–14,000× *g* rcf for 2 min, and the supernatant was discarded. To ensure thorough cleaning, the centrifugation and supernatant removal steps were repeated once more [14].

#### 2.1.3. Protein Extraction and Isolation Process

Ultimately, a small amount of the protein precipitate (approximately 3 mm^3^) was transferred to a 1.5 mL tube, and 200 μL of an organic solvent (50% acetonitrile, 47.5% water, 2.5% trifluoroacetic acid; Merck, Darmstadt, Germany) was added. The solution was homogenised by stirring with a spatula and then vortexed for 10–20 s. Following this, the mixture was centrifuged at 12,000–14,000× *g* rcf for 2 min. Subsequently, 1 µL of the supernatant was applied to the analysis plate. After drying, 1 µL of HCCA matrix solution (Bruker Daltonik GmbH, Bremen, Germany) was added [41,44].

### 2.2. Mass Spectrometry Analysis

Detailed methodological framework regarding MALDI-TOF MS analysis: Milk samples were analysed using a Microflex LT™ MALDI-TOF MS instrument (Bruker Daltonik GmbH, Bremen, Germany) equipped with a 337 nm nitrogen laser (60 Hz) in positive linear mode with a 20 kV acceleration voltage. The reflection mode enhanced ion transmission for large biomolecules (i.e., caseins), and the laser was set at 40–45% power to optimize ionisation while minimizing fragmentation. Mass spectra were acquired over an *m*/*z* range of 3000–10,000 Da, capturing key protein and peptide signatures of bovine, ovine, and caprine milk at an optimized resolution of 8000–12,000 FWHM for precise peak differentiation. Each spectrum was generated from 500 laser shots and averaged over five acquisitions per sample to mitigate inhomogeneities and matrix crystallization variations. For robust statistical power in machine learning-based classification, each sample was analysed in triplicate, yielding at least 24 valid spectra. Internal calibration using a bacterial test standard (BTS, Bruker Daltonik) was performed before each session, ensuring mass accuracy within ±200 ppm and consistent peak alignment. Rigorous spectral quality control—including normalization, baseline subtraction, and peak alignment—ensured consistency across biological replicates [14,37,44].

Following casein extraction, milk samples were analysed using manufacturer-recommended software: flexControl for measurement, flexAnalysis for data quality control, and Biotyper OC for MSP generation. Major spectral peaks were identified by comparing their *m*/*z* values to known molecular weights of casein proteins (from the literature and calibration standards), confirming that our method successfully targeted the casein fraction. A new analysis run was initiated using the AutoXecute Run Editor, which enabled the preparation of an automatic analysis based on defined specifications to create new entries in a custom database. Each MALDI-TOF plate position was assigned a sample designation in the Sample Name cell. In the AutoXecute Method cell, factory settings were applied via the MBT_AutoX basic method, in strict accordance with the manufacturer’s guidelines. Once the parameters were set, the analysis was programmed for each position accordingly.

To examine milk samples following the casein extraction process, the manufacturer-recommended software was utilised: flexControl (version 3.4) for measurement, flexAnalysis (version 3.4) for data quality control, and Biotyper OC (version 3.1) for MSP generation. Initially, a new analysis (new run) was created by opening the AutoXecute Run Editor. This tool facilitated the preparation of a new automatic analysis based on the desired specifications to generate new entries for a custom database.

In the card Sample Name cell, each position on the MALDI-TOF plate was labelled according to the corresponding sample designated for analysis. In the AutoXecute Method cell, in accordance with the manufacturer’s recommendations, the factory setting was selected utilizing the basic method of the MBT_AutoX analyser. After selecting the parameters in the specified cells, the analysis was programmed for each position accordingly.

During the QC of the sample spectra, the spectrum files were visually normalised and zoomed in, allowing for the identification and removal of any invalid spectra, such as flatlines, outliers in the “sweet spot” region, and other anomalies.

Subsequently, the approved spectra were loaded into the program using the “Add Spectra” option. The program saved these spectra, which then appeared as unregistered MSPs within the taxonomy tree. To register these MSPs, a new folder was created specifically for this database. Finally, the “Start Taxonomy Tree Editor” option enabled the registration of the MSPs within the newly created database.

**Figure 3:**
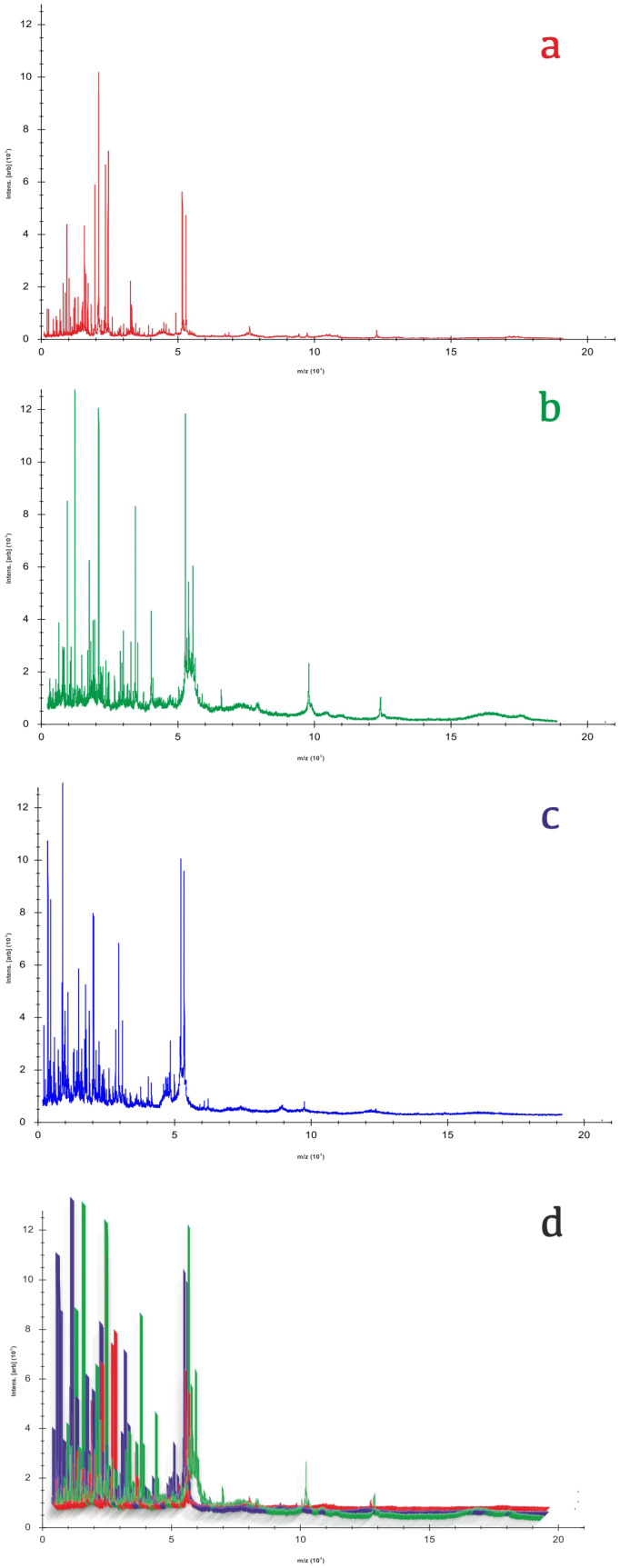
Characteristic spectral profiles of milk samples obtained from bovine (**a**), ovine (**b**), caprine (**c**) sources and (**d**) overlay of the three species’ spectra (Colours correspond to species as follows: red = bovine, green = ovine and blue = caprine).

Figure 3 displays representative spectra of bovine, ovine, and caprine milk, highlighting distinct differences in their spectral profiles.

### 2.3. Data Extraction

The data extraction was performed by the manufacturer’s recommended software FlexControl (version 3.4). Prior to exporting, we flattened (smoothing) and normalised (subtraction) every spectrum within the program. The processed spectra were then exported in .xlsx format. Each spectrum was included in a separate excel file, identified by the name of the farm where the milk was produced. Thus, we ensured that our data would be easily accessible for a variety of analytical and machine learning tasks, facilitating a more straightforward development of our classification model.

### 2.4. Data Preprocessing

To organise our data, we utilised python (version 3.10.13) to automatically extract all spectra from each Excel file, ensuring they were labelled with the corresponding farm name and the correct animal origin. In total we gathered 4594 spectra from 51 different farms. For optimal spectral quality, we selected spectra with an intensity of ≥1000, a signal-to-noise ratio (S/N) of ≥5, and a resolution of ≥400. Any row containing at least one value below these thresholds was replaced with NaN. Subsequently, all NaN values were removed from the spectra data, leading to a nx8 datasets from each run, which were organised into separate folders for further analysis. Our 8 features are mass to charge ratio (*m*/*z*), time, intensity, S/N, resolution, area, relative intensity and Full-width-at-half-maximum (FWHM). Each run represents an analysis conducted on the mass spectrometer with a different orientation of the sample. Within each folder, we computed the differences between the masses from all pairs of spectra files. This process allowed us to determine the closest masses across several spectra, which were then averaged to a final vector with dimensions of 1 × 8. Due to the nature of the spectra, it is important to note that the data was scaled using a Robust Scaler (1) to ensure proper normalization while accounting for outliers, as they are the primary focus of our analysis [45].

(1) $$x^{\prime }=\frac{x-Median\left(x\right)}{IQR\left(x\right)}$$

This final vector was generated repeatedly, n times, corresponding to the total number of rows from the different runs. Each vector consists of 8 numerical values representing selected peaks extracted from the mass spectrometry analysis. Ultimately, all the resulting datasets were merged to create a final dataset with the dimensions 109,467 × 8, which was used for the development of our neural network. This includes 16,018 vectors representing bovine milk, 38,394 vectors for goat milk, and 55,055 vectors for sheep milk, each with 8 dimensions corresponding to these selected peaks.

### 2.5. Neural Network Model Development

For the development and training of our neural network, we used Tensorflow (version 2.10.0). To ensure clear and effective classification of milk types, we chose three categorical labels, namely Bovine_Milk, Goat_Milk and Sheep_Milk. These labels were converted into binary vectors using One Hot Encoding (OHE) to facilitate multi-class classification. This encoding transforms each label into a vector where a single element is active (1) to represent the class, while all other elements are inactive (0), making the data compatible with the categorical cross-entropy loss function used in training (Bovine_Milk (OHE: [1, 0, 0]), Goat_Milk (OHE: [0, 1, 0] and Sheep Milk [0, 0, 1]). Then, we employed a k-fold cross-validation strategy to enhance the robustness of our model. K-fold cross-validation involves splitting data into k equal subsets, every time using only one subset as a test set, while the remaining parts (k-1) serve as the training set. In our methodology, we split our dataset into 10 equal subsets 10 times, each time taking one subset as a test set (10-fold cross validation). By definition, each iteration is independent, with a new test set in every run [46].

However, because of the imbalance in the dataset, these portions could result in poor representation of the Bovine class (minor class). Addressing this issue was crucial, and thus, we applied class weights during training, giving higher importance to the Bovine class, and secondarily to the Goat class (Table 1). Robust scaling was applied after splitting the dataset to further enhance model performance while avoiding data leakage.

**Figure 4:**
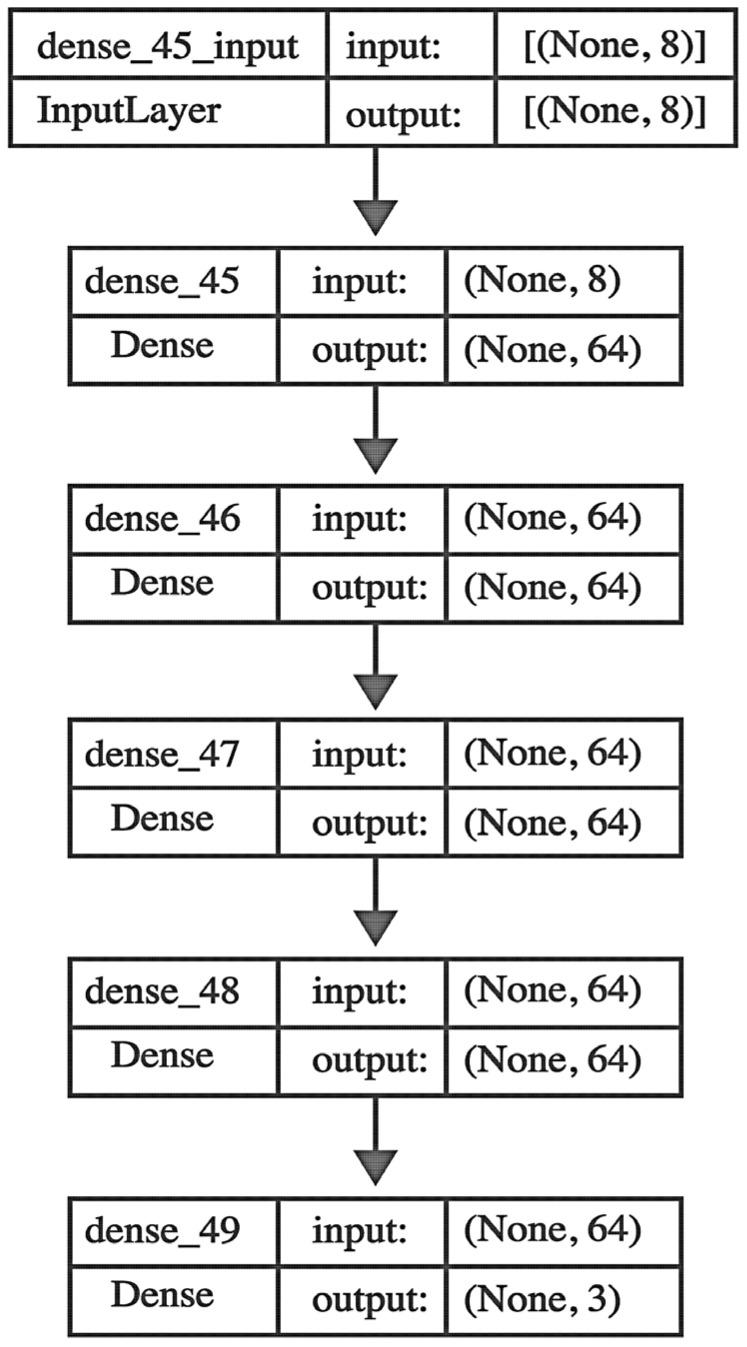
Our model architecture consists of an input layer with 8 nodes, followed by four hidden layers, each containing 64 nodes. The output layer is composed of 3 nodes. Arrows between layers represent the fully connected, weighted links through which information flows forward from one layer to the next.

Following these steps, the processed data was then fed into our neural network model, BoSheeGO. Our approach follows the classic Feed Forward Neural Network (FNN) model architecture, with an input layer that accepts 8-dimensional feature vectors, corresponding to the spectral peaks of the milk samples. Then, four fully connected layers (hidden layers), each consisting of 64 neurons, follow the input layers. On each hidden layer we employed the ReLU (Rectified Linear Unit) activation function (2), enabling the network to capture complex, non-linear relationships within the data (Figure 4). After extensive research into the optimal number of nodes and hidden layers, we arrived at the above architecture to enable fast training on complex patterns without requiring significant computational power. This structure also allowed the neural network to avoid overfitting, demonstrating strong generalisability.

(2) $$f\left(x\right)=\max\left(0,x\right)$$

The final layer consists of 3 neurons with a softmax (3) activation function, where $x_{i}$ is the model’s output for the *i-th* class before applying the softmax function, *n* is the total number of classes (3 in our case), and the denominator normalises the model’s outputs for the three classes to scale [0,1].

(3) $$s\left(x_{i}\right)=\frac{\exp\left(x_{i}\right)}{\sum_{j=1}^{n}\exp\left(x_{j}\right)}$$

This architecture outputs a probability distribution across the three classes—Bovine_Milk, Goat_Milk, and Sheep_Milk—allowing for the classification of milk types. The model was optimised for the milk-type classification using the adam optimizer, known for its efficiency in finding optimal parameters. To handle the multi-class nature of the problem, categorical cross-entropy loss (4) was employed to measure the model’s prediction accuracy. Let $y_{i}$ and $\hat{y_{i}}$ represent the true label and the predicted probability for the *i-th* class of the model, respectively, while *C* means the total number of classes (3 in our case). The result is the overall loss determined by aggregating the contribution from each class. The performance was evaluated based on the accuracy, precision, recall, and AUC metrics. The training was conducted over 25 epochs with a batch size of 32 to achieve a balance between computational efficiency and model convergence.

(4) $$H\left(y,\hat{y}\right)=-\sum_{i=1}^{c}y_{i}\cdot \log\left(\hat{y_{i}}\right)$$

## 3. Results

Figure 3 illustrates a dendrogram of the entries in the MALDI-TOF libraries, highlighting the clusters of spectra associated with the three ruminant species. This representation enables the rapid identification of clusters, facilitating further investigation into the distinct features of each species. To support this analysis, we developed the proposed feedforward neural network (FNN) and assessed the model’s robustness and generalisation using various metrics during a 10-fold cross-validation (Table 2).

### 3.1. Loss and Accuracy

Close convergence is shown by the depicted loss curves (Appendix A.1. Supplementary Data 1) for both training and testing, indicating that the model was successful in identifying the underlying patterns in the data. Because of the stochastic nature of the training process, a small variation in the test loss relative to the training loss was seen, which is typical and expected. With an average loss of 0.43 ± 0.005 for the training dataset and 0.53 ± 0.015 for the test dataset over all 10 folds, the model was able to reduce the difference between the expected probabilities and actual labels. This loss value represents the efficiency of the model in fitting the training data and generalising to new data.

The average accuracy (5) was 0.76 ± 0.003 for the training dataset and 0.75 ± 0.007 for the test dataset. The model’s ability to accurately sort the majority of milk samples into the appropriate categories—Bovine_Milk (Class 0), Goat_Milk (Class 1), and Sheep_Milk (Class 2) is demonstrated by its high accuracy. The consistency in accuracy across different folds of cross-validation demonstrates the model’s reliability and robustness in diverse data scenarios.

(5) $$\mathrm{Acc}=\frac{TP+TN}{TP+FN+TN+FP}$$

### 3.2. Precision and Recall

Precision and recall (6) are critical metrics for evaluating the model’s performance, particularly when dealing with imbalanced datasets. Precision measures the proportion of true positive classifications among all positive predictions, while recall measures the percentage of true positives among all actual positive cases.

The average precision across all folds is 0.77 ± 0.003 for the training dataset and 0.76 ± 0.007 for the test dataset, while the average recall is 0.75 ± 0.004 and 0.74 ± 0.009, respectively. These values reflect our model’s efficiency in reducing false positives as well as false negatives.

(6) $$\mathrm{Pre}=\frac{TP}{TP+FP}\;\mathrm{Rec}=\frac{TP}{TP+FN}$$

### 3.3. ROC and AUC

For each fold in the 10-fold cross-validation, ROC curves were plotted to visually evaluate how well the model distinguished between the various types of milk (Appendix B.1. Supplementary Data 2). The ROC curve plots the True Positive Rate (TPR) against the False Positive Rate (FPR) (7) across different classification thresholds, illustrating how well the model distinguishes each milk type from the others, aiding the visualisation of the AUC metric.

(7) $$\mathrm{TPR}=\frac{TP}{TP+FN}\;\mathrm{FPR}=\frac{FP}{FP+TN}$$

The AUC metric provides a summary measure of the model’s performance across all classification thresholds. It evaluates the ability of the model to distinguish between classes, with values closer to 1 indicating better performance. The model achieved an average AUC value of 0.90 ± 0.023 for the training dataset and 0.90 ± 0.018 for the test dataset across all classes. Also, by using the One vs Rest method [47], the AUC of each class can be seen in for each fold, which consolidates that the NN performs well in both sensitivity and specificity, across various thresholds. These results indicate that the model is well-optimised, achieving a satisfactory trade-off between training performance and generalisation to test data. Our findings suggest that the model is robust and can potentially perform well in practical deployment scenarios.

Furthermore, as a preliminary analysis, we tested five mixtures at a 50/50 ratio of goat and sheep milk, with the results presented in Table 3. For future work, we plan to expand our dataset by adding mixtures with varying proportions of goat, sheep, and bovine milk.

## 4. Discussion

Milk is a complex matrix of organic compounds, primarily fats and proteins. Each species’ milk protein profile is distinctive yet inherently complex, making conventional analysis labour-intensive and prone to uncertainty. Given the molecular diversity of milk, detecting adulteration by identifying chemical correlations requires advanced analytical techniques coupled with sophisticated data interpretation. These methods must address the intricacies of milk composition to yield transparent, accurate, and reliable outcomes [47]. According to EU legislation (2018/150), the official method for detecting the admixture of bovine milk in milk from other species is based on the isoelectric focusing (IF) of γ-caseins—a laborious approach that offers limited results [32,48,49].

MALDI-TOF MS has emerged as a transformative technology in food analysis by delivering rapid, accurate, and cost-effective species identification. Nevertheless, it is by no means the sole mass spectrometry (MS) platform driving innovation; LC–MS/MS and other advanced MS approaches have also profoundly reshaped food authenticity testing. For instance, von Bargen et al. (2014) [10] demonstrated how LC–MS/MS exhibits high specificity in detecting horse and pork adulteration in processed foods, whereas Tehrani et al. (2024) [35] illustrate the enhanced scope and reliability achieved when MALDI-TOF MS is coupled with complementary MS-based strategies for detecting and quantifying milk adulterants. Beyond stand-alone MS techniques, omics-based tools—proteomics, genomics, and metabolomics—broaden our molecular-level understanding of food systems and bolster the detection of even subtle adulteration. Behkami et al. (2019) [50] and Li et al. (2024) [51] highlight multi-omics frameworks that accurately classify milk and identify adulteration, while Zhu et al. (2024) [52] uses label-free proteomics to unveil nuanced compositional differences among various milk species. Similarly, Di Luca et al. (2023) [53] show how label-free LC–MS proteomics can discriminate product variations across distinct breeds, emphasizing the granularity achievable through integrated omics pipelines. Taken together, these evolving technologies form a multi-disciplinary ecosystem in which MALDI-TOF MS—when deployed alongside LC–MS/MS and omics-driven methods—amplifies the speed, resolution, and confidence in food authentication processes. This synergy is increasingly vital for ensuring consumer safety, maintaining product integrity, and advancing real-time detection to meet emerging challenges in global food supply chains.

MALDI-TOF MS exhibits exceptional analytical capability for detecting proteins and peptides within complex molecular mixtures [37]. It offers several technical advantages: minimal sample preparation, negligible sample volume requirements, and low reagent costs, making it highly efficient for processing large numbers of samples within short timeframes [2]. This technique has been successfully applied and validated for species identification in various matrices, including muscle meat, scallops, seafood, fish, and edible insects [21,22,23,24]. Its efficiency and cost-effectiveness render it a superior alternative to traditional electrophoretic and chromatographic methods, which are typically associated with higher operational costs and longer processing times [54,55].

In milk protein analysis, MALDI-TOF MS facilitates the detection and differentiation of specific proteins—such as caseins, α-lactalbumin, and β-lactoglobulin—based on their unique molecular masses. Variations in the amino acid sequences of these proteins enable discrimination between milks from different species, highlighting the method’s precision and versatility [37]. For example, Calvano et al. (2012) demonstrated that the intensity of the MALDI-TOF MS signal for characteristic proteins in bovine and caprine milk was proportional to the level of adulteration [28]. Girolamo et al. (2014) detected the adulteration of donkey and caprine milk with bovine milk at a precision level of 0.5% [29]. In another study, Nicolaou et al. (2011) analysed binary and ternary mixtures of bovine, ovine, and caprine milk by examining the spectra of specific proteins (α-lactalbumin, β-lactoglobulin, and γ2- and γ3-casein) and achieved adulteration detection with maximum error percentages ranging from 2% to 13%, depending on the mixture [30]. Lastly, Rysova et al. (2022) reported maximum errors of 11.2% for caprine milk and 12% for ovine milk [56].

Accurately measuring adulteration in milk mixtures is essential yet challenging, due to the numerous factors influencing milk composition. Variability in breed, parity, season, lactation stage, geographical region, husbandry practices, and farming systems can significantly affect milk characteristics and complicate detection. Advanced analytical techniques, such as MALDI-TOF MS, can identify these compositional variations. However, without a reliable and well-curated reference dataset, the results may be ambiguous or misleading. To accurately determine correlations and cluster patterns, the datasets must be comprehensive, highly reliable, and meticulously curated. Weis et al. (2020) emphasize that for machine learning techniques to achieve their full potential, three critical challenges must be addressed: limited sample dataset sizes, lack of external validation, and the low reproducibility of results [57].

Large datasets of milk protein spectra, particularly those acquired via MALDI-TOF mass spectrometry, remain limited [35,58]. A primary objective of this study was to establish a comprehensive collection of protein spectra from bovine, ovine, and caprine milk, including samples from key dairy regions such as Greece. This dataset is intended to serve as a valuable resource for machine learning applications and as a robust reference standard for the dairy industry. Despite the meticulous and labour-intensive effort involved, further dataset expansion is necessary to tackle the growing issue of ovine and caprine milk adulteration—a concern that is becoming increasingly significant in the European Union and in Mediterranean countries [59].

In recent years, consumer preferences have increasingly favoured milk and dairy products from small ruminants, driven by culinary traditions, perceived health benefits, and sustainable farming practices [60]. This shift has raised the market value of ovine and caprine milk relative to cow milk, and cases of adulteration—where lower-cost cow milk is mixed with higher-value small ruminant milk—have emerged, challenging industry integrity. This study focuses on regions such as Greece, which plays a key role in the small ruminant dairy sector, particularly in the production of Protected Designation of Origin (PDO) products. As of 2023, Greece accounted for 25.8% of the EU’s goat population and 13.7% of its sheep population [61], and it produces twenty-one PDO cheeses, underscoring the cultural and economic importance of its dairy industry. These factors highlight the need for precise analytical methods to verify the authenticity of small ruminant milk and its derivatives. By employing advanced spectrometric techniques and developing a comprehensive dataset, this study aims to establish a solid foundation for quality control, traceability, and the application of machine learning to address milk adulteration challenges.

The complexity and volume of raw spectral data require specialized algorithms for pattern recognition and cluster analysis [55]. Machine learning techniques are particularly effective in this context, as they can uncover statistical correlations and non-linear interactions within the data. Consequently, these methods have the potential to reveal previously unrecognized evidence embedded in MALDI-TOF spectra [57].

The lack of extensive prior studies on milk samples has highlighted a critical gap in the field and underscored the need for novel research in this area. The development of a comprehensive dataset of milk samples represents a significant step forward, with the potential to advance ongoing research while shaping agricultural practices and food quality protocols. To address this gap, an established protocol [14] was utilized to generate a large-scale dataset of milk samples. This ambitious task not only marked a major milestone for the study but also laid the groundwork for more detailed investigations. Managing and analysing such an extensive dataset required meticulous planning, advanced data processing capabilities, and robust analytical strategies.

The extensive dataset enhanced statistical power, allowing for the precise identification of subtle, species-specific spectral patterns. However, the large volume of data also presented challenges in storage, management, and analysis, necessitating innovative solutions to maintain the integrity and reliability of the study’s findings.

The inability to manually edit the parameters of the MALDI-TOF analysis software could potentially impact the specificity and sensitivity of the analysis. In addition, the need for a quick and inexpensive protocol for the analysis of milk samples was a driving force behind the methodological choices in this study. For this reason, in the current study, we analysed milk samples from bovine, ovine, and caprine species by examining their mass spectrometry profiles, with a specific focus on casein masses. Utilizing Bruker’s MALDI-Biotyper system, the study leverages mass spectrometry features such as *m*/*z*, time, intensity, signal-to-noise ratio (SN), resolution, area, relative intensity, and full width at half maximum (FWHM) to train a neural network model. The ANN developed in this study consists of eight input features derived from the mass spectrometry data, 64 nodes in each of four hidden layers, and three nodes in the output layer corresponding to the three species being classified. Our model demonstrates robust performance across five evaluation metrics (Table 4). Specifically, it achieved an AUC of 0.90 ± 0.023 for the training dataset and 0.90 ± 0.018 for the test dataset, indicating a high capacity for distinguishing between the different milk samples. Precision was similarly high, recorded at 0.77 ± 0.003 for the training dataset and 0.76 ± 0.007 for the test dataset, reflecting the model’s reliability in minimizing false positives. Recall values of 0.75 ± 0.004 and 0.74 ± 0.009 for the training and test datasets, respectively, further enhance the model’s ability to correctly identify positive cases. Additionally, the loss values of 0.43 ± 0.005 for training and 0.53 ± 0.015 for testing underscore the model’s consistency in learning without overfitting. Finally, the accuracy of the model at 0.76 ± 0.003 for training and 0.75 ± 0.007 for testing highlights its overall effectiveness in milk classification. Therefore, our novel approach aids in detecting species-specific spectral patterns, facilitating the accurate identification of milk composition and the detection of potential impurities.

The milk origin identification research presented in the current study represents a significant advancement in the field of food authentication, particularly in the dairy industry. This innovative approach combines cutting-edge proteomic technologies with artificial neural networks (ANNs) to address critical challenges in milk origin determination, adulteration detection, and quality assurance. One of the most distinctive features of this study is its comprehensive multi-species classification approach. Unlike previous studies that have often focused on binary classification or single-species identification [50], this research simultaneously classifies milk samples from three different species: bovine, ovine, and caprine. This multi-class approach provides a more realistic and applicable solution for the dairy industry, where mixed-species adulteration is a common concern. The ability to distinguish between multiple species in a single analysis represents a significant advancement over traditional methods and addresses a critical need in the field of milk authentication [51].

The dataset used in this study is unprecedented in the field of milk origin identification. It includes 648, 1554, and 2392 spectra from bovine, caprine, and ovine milk, corresponding to 16,018, 38,394, and 55,055 eight-dimensional vectors, respectively. This extensive dataset increases the statistical power of our analysis, enabling more precise identification of species-specific spectral patterns [45]. Moreover, the ability to manage and analyse such a large volume of data underscores the robustness and scalability of our approach, setting a new benchmark for large-scale proteomic analysis in food authentication.

Integrating MALDI-TOF MS data with artificial neural networks enhances pattern recognition for milk origin identification. By leveraging ANNs, the model can detect subtle, species-specific patterns in mass spectrometry profiles that might be overlooked by traditional analytical techniques or human analysis. This approach is especially valuable for identifying sophisticated forms of adulteration that may not be detected by conventional testing methods [62].

The methodology employed in this study demonstrates several innovative aspects: First, it introduces a rapid and cost-effective protocol for analysing milk samples, addressing the industry’s need for efficient and accessible authentication methods [44]. Second, by focusing on casein masses in the mass spectrometry profiles, the approach leverages unique protein signatures for targeted species identification. Notably, the protein extraction was optimised for caseins with acetic acid precipitation, enriching casein proteins for analysis. The MALDI-TOF MS spectra featured major peaks corresponding to known casein protein masses, confirming successful isolation of the casein fraction. Furthermore, we observed that each species’ spectrum contained particular peak patterns, forming a spectral ‘fingerprint’ for each milk type. Third, the model incorporates multiple features—including *m*/*z*, time, intensity, signal-to-noise ratio (SN), resolution, area, relative intensity, and full width at half maximum (FWHM)—to enable a comprehensive analysis of the spectral data. Finally, the development of a custom neural network tailored to process complex MALDI-TOF MS data represents a novel application of machine learning in food authentication [34].

This study addresses several critical challenges in the dairy industry. The model’s high accuracy in distinguishing among milk samples from different species offers a robust tool for detecting adulteration, a persistent issue in dairy production. By providing a rapid and reliable method for species identification, the approach enhances quality control processes during dairy production and processing. Moreover, the ability to quickly and accurately determine milk origin contributes to improved food safety measures, reducing the risk of distributing mislabelled or adulterated products [62].

The innovative aspects of this work have significant implications for future research and broader applications in food authentication. The methodology developed herein can be adapted to authenticate a range of food products beyond dairy, thereby enhancing overall food safety and quality assurance. Additionally, the approach is readily integrable with existing quality control systems in the dairy industry, bolstering product integrity and consumer trust. With its high accuracy and reliability, this method shows promise as a potential standard for regulatory compliance in milk authentication, addressing the need for standardized protocols [63].

As the dairy industry continues its digital transformation, the application of machine learning and artificial intelligence to spectroscopic and proteomic data analysis is expected to become increasingly prevalent. These advanced technologies offer new insights and efficiencies in milk authentication and align with broader industry trends, including the integration of robotics and automation in dairy farming, the adoption of wearable technology for animal health monitoring, and the use of data analytics to optimize operations [64].

## 5. Conclusions

The present study addressed several key challenges: limited research on regional milk samples, the creation and management of a comprehensive dataset, constraints in customizing MALDI-TOF parameters, and the need for a rapid, cost-effective analysis protocol. The proposed methodology successfully overcame these obstacles, representing a significant advancement in milk analysis.

A robust artificial neural network (ANN) model was developed using a unified dataset of 109,467 samples with eight features derived from casein spectral data. This model effectively classified milk into three distinct categories—bovine, goat, and sheep—with strong evaluation metrics. Notably, generalization between training and testing data is demonstrated by an AUC of approximately 0.90, a precision of 0.76, a recall of 0.74, a loss of 0.53, and an accuracy of 0.75, underscoring the model’s robustness.

Although the findings of this study are promising, it is important to emphasize that BoSheeGo is limited to casein spectra, restricting its application to analyses involving only this type of protein. Additionally, our dataset includes only Epirus samples, highlighting the need for a broader geographical distribution, seasonal variations, and diverse processing methods to improve the model’s generalizability and applicability. In addition, exporting spectral data as images could contribute to the creation of advanced neural network architectures, such as convolutional neural networks (CNNs), providing solutions to the adulteration problem from a different scope.

In future research, advanced proteomic analyses integrated with robust genetic assays can significantly enhance both the reliability and breadth of milk authentication protocols, providing a powerful fail-safe system that reinforces consumer and regulatory confidence. As datasets expand to encompass broader geographical and seasonal variations, parallel genetic verifications will become increasingly pivotal for refining and confirming MALDI-TOF MS–based classifications, ultimately setting a higher standard for dairy product integrity.

## Figures and Tables

**Table 1:** Class weights: Used to adjust the classification of the neural network in response to data imbalance. Each weight corresponds to a specific class, improving the model’s ability to accurately classify underrepresented categories.

Folds	0 (Bovine_Milk)	1 (Goat_Milk)	2 (Sheep_Milk)
1	2.275814276	0.95086429	0.662724759
2	2.273923279	0.950781702	0.662925431
3	2.279447491	0.95102951	0.662337138
4	2.272978959	0.950341475	0.663219969
5	2.285793833	0.949407343	0.662591046
6	2.275183594	0.9495446	0.663420941
7	2.270621586	0.951690961	0.662764884
8	2.281687858	0.949636612	0.662825119
9	2.278363628	0.950406128	0.662731486
10	2.28629444	0.950131158	0.66219695

**Table 2:** Model Statistics averaged across 10-fold cross-validation.

	Average	Standard Deviation
Train Loss	0.43	0.005
Test Loss	0.53	0.015
		
Train Categorical Accuracy	0.76	0.003
Test Categorical Accuracy	0.75	0.007
		
Train Precision	0.77	0.003
Test Precision	0.76	0.007
		
Train Recall	0.75	0.004
Test Recall	0.74	0.009
		
Train AUC	0.90	0.023
Test AUC	0.90	0.018

**Table 3:** Results of the evaluation of our model in 50% goat and 50% sheep milk mixtures. The error is calculated as the deviation from a 50–50 classification ratio in our predictions, and the average of these deviations is used as the overall error metric.

Sample	Bovine	Goat	Sheep	Error
1	0	0.6	0.4	±0.1
2	0	0.57	0.43	±0.07
3	0	0.55	0.45	±0.05
4	0	0.6	0.4	±0.1
5	0	0.48	0.51	±0.03
Average				±0.07

**Table 4:** Confusion matrix across the three milk classes: Bovine (Class 0), Goat (Class 1), and Sheep (Class 2). The diagonal elements indicate the correctly classified spectra, while the off-diagonal values represent the model’s misclassifications. The FNN achieves an overall test accuracy of 0.75, with Class 0 showing the highest recall (0.93), while Class 2 has the highest precision (0.81). Class 1 has the lowest precision (0.69) and a recall of 0.77.

		Predicted Class		
		Bovine (Class 0)	Goat (Class 1)	Sheep (Class 2)
True Class	Bovine (Class 0)	14,846	240	932
	Goat (Class 1)	855	29,563	7976
	Sheep (Class 2)	4531	13,021	37,503

**Figure A1:**
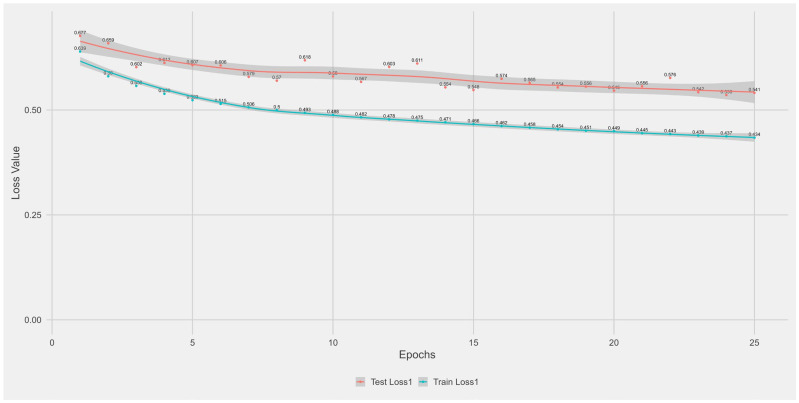
Training and test loss curve for k = 1.

**Figure A2:**
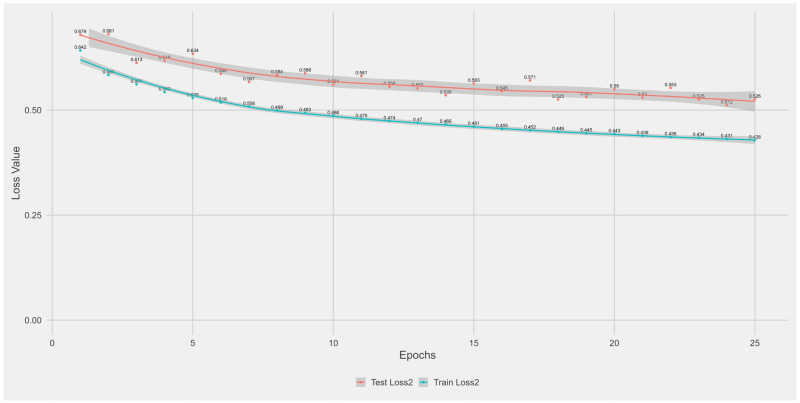
Training and test loss curve for k = 2.

**Figure A3:**
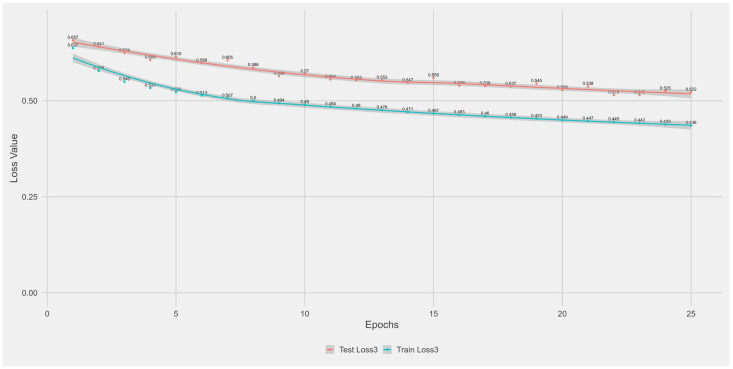
Training and test loss curve for k = 3.

**Figure A4:**
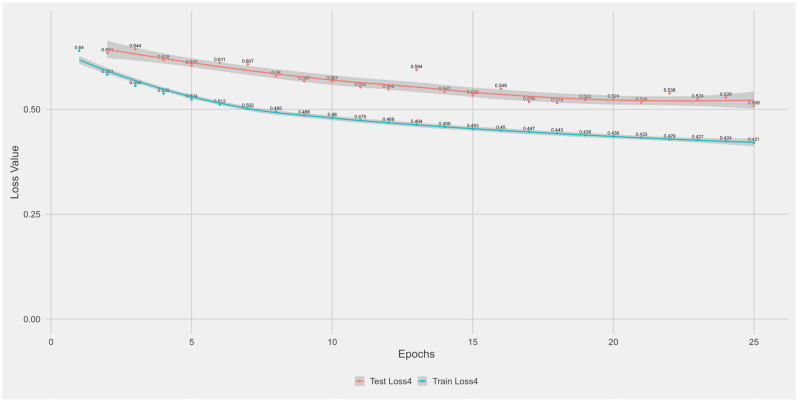
Training and test loss curve for k = 4.

**Figure A5:**
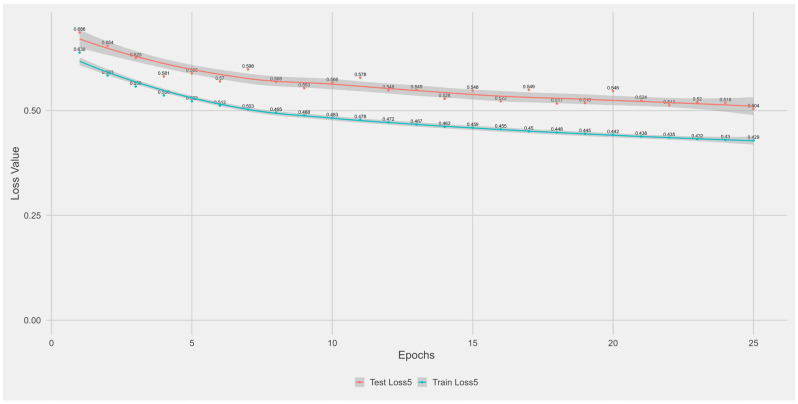
Training and test loss curve for k = 5.

**Figure A6:**
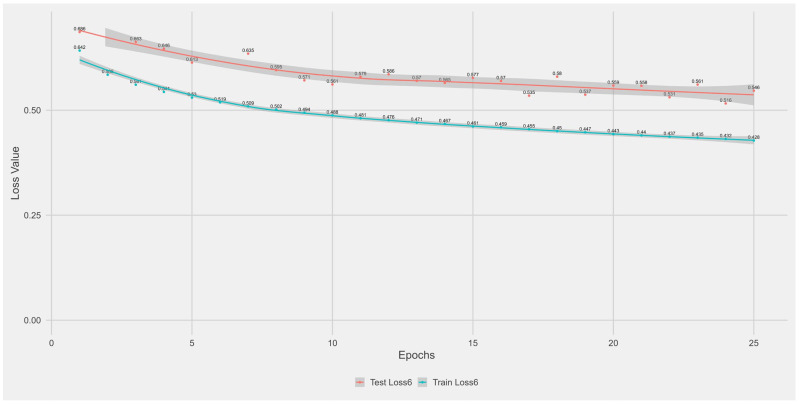
Training and test loss curve for k = 6.

**Figure A7:**
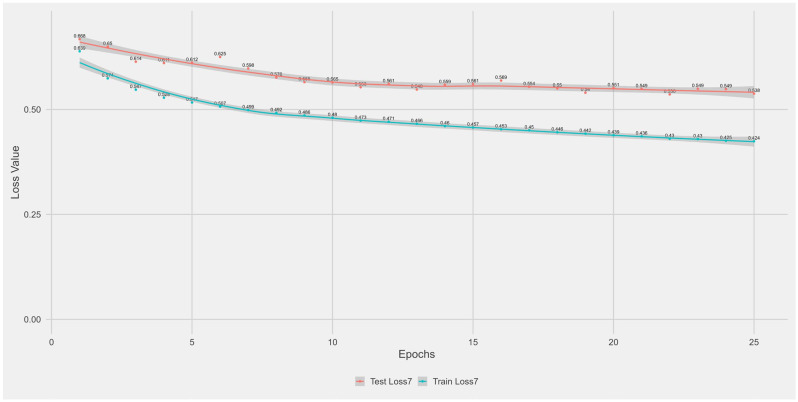
Training and test loss curve for k = 7.

**Figure A8:**
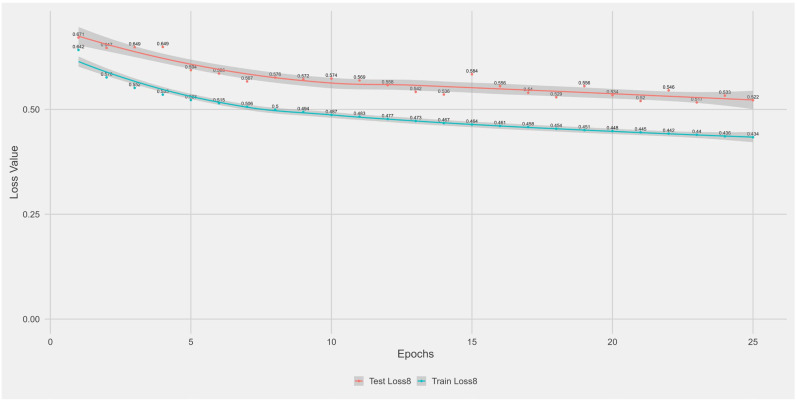
Training and test loss curve for k = 8.

**Figure A9:**
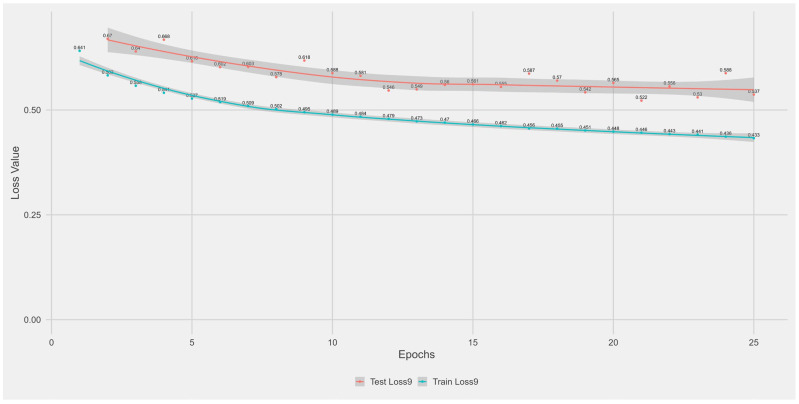
Training and test loss curve for k = 9.

**Figure A10:**
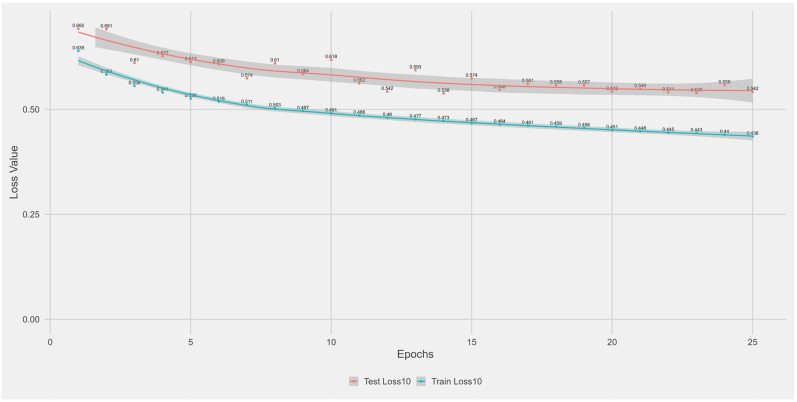
Training and test loss curve for k = 10.

**Figure A11:**
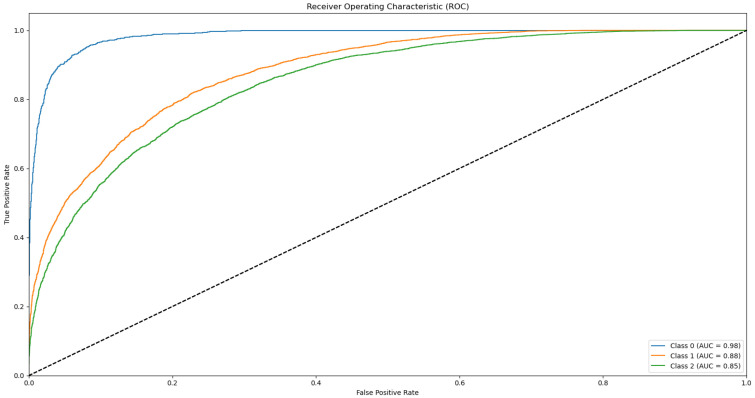
ROC curve for k = 1.

**Figure A12:**
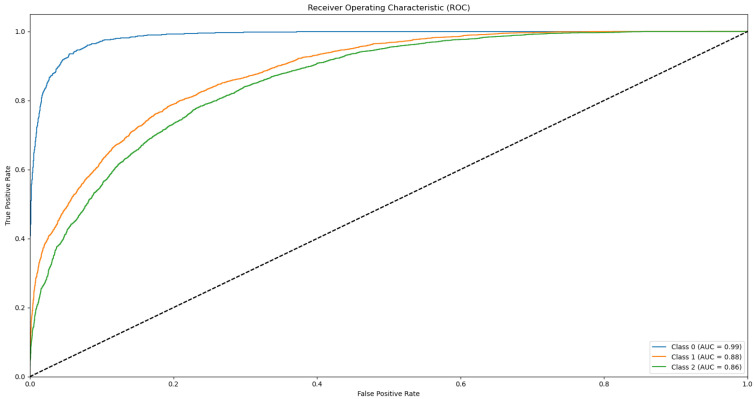
ROC curve for k = 2.

**Figure A13:**
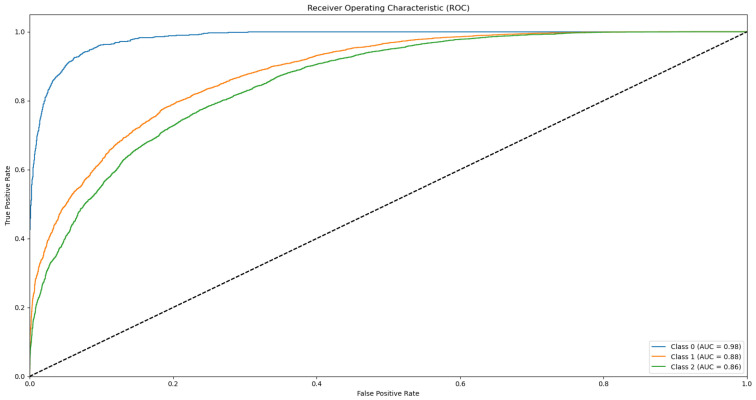
ROC curve for k = 3.

**Figure A14:**
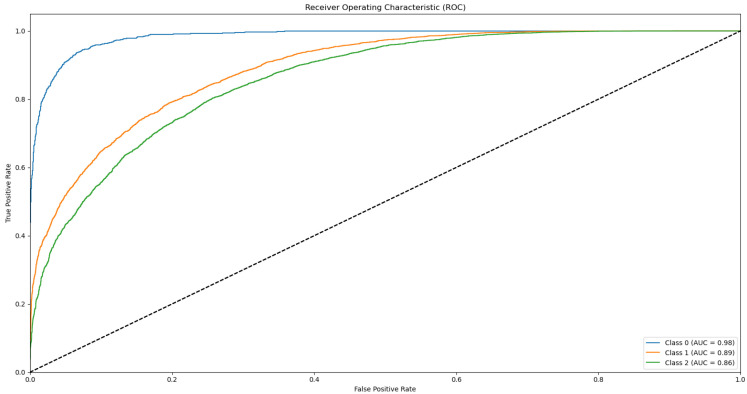
ROC curve for k = 4.

**Figure A15:**
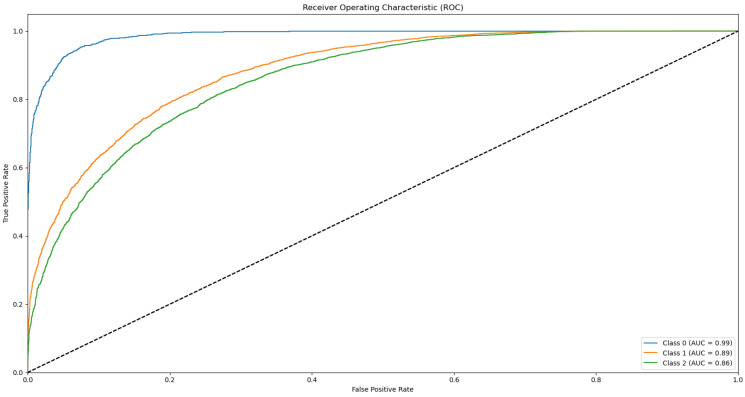
ROC curve for k = 5.

**Figure A16:**
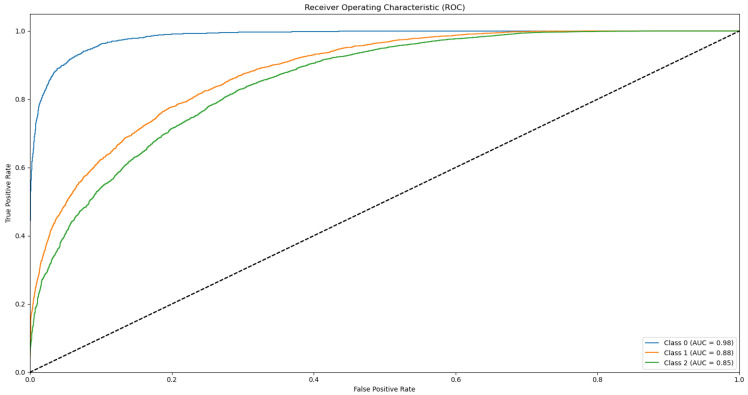
ROC curve for k = 6.

**Figure A17:**
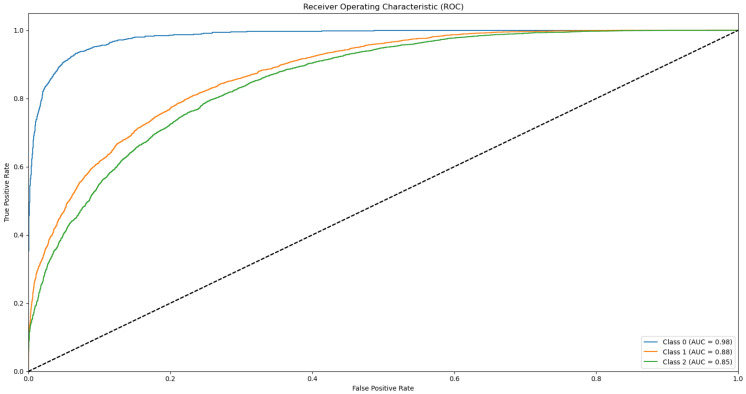
ROC curve for k = 7.

**Figure A18:**
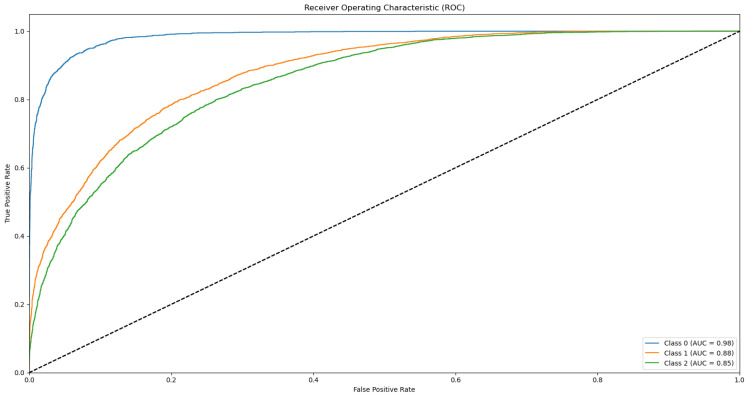
ROC curve for k = 8.

**Figure A19:**
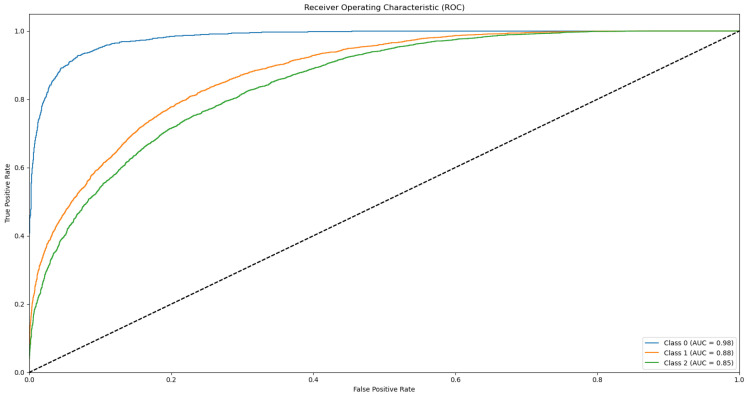
ROC curve for k = 9.

**Figure A20:**
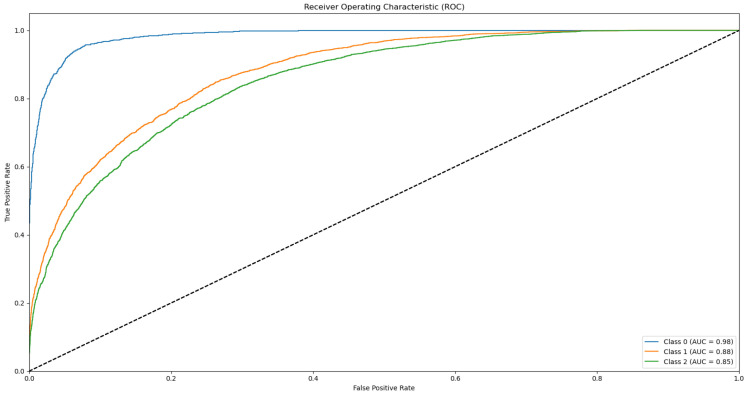
ROC curve for k = 10.

## Data Availability

The data that support the findings of this study are openly available in Zenodo at https://doi.org/10.5281/zenodo.15024170.

## Abbreviations

The following abbreviations are used in this manuscript:

MS	Mass Spectrometry
MALDI—TOF MS	Matrix-assisted laser desorption/ionization-time of flight mass spectrometry
MSP	Main Spectral Profile
CE	Capillary Electrophoresis
LC	Liquid Chromatography
UA	Ultraviolet Absorption
FNN	Feedforward Neural Network
BTS	Bacterial Test Standard
QC	Quality Control
*m*/*z*	Mass to Charge ratio
SN	Signal to noise ratio
FWHM	Full-width-at-half-maximum
OHE	One Hot Encoding
ReLU	Rectified Linear Unit
TPR	True Positive Rate
FPR	False Positive Rate
AUC	Area under the Receiver Operating Characteristic Curve
ROC	Receiver operating characteristic

## Appendix A

### Appendix A.1. Supplementary Data 1: Loss Function per Epoch

In Appendix A is presented the training and test loss curves across 25 epochs for each of the 10 folds. These plots provide insights into the model’s learning progress and its generalisation capability across different epochs. Each of the following 10 plots shows:

- The training loss (solid blue line), reflecting the model’s error on the training data after each epoch.
- The test loss (solid orange line), indicating how well the model generalises to unseen data.
- The grey area is the Confidence interval.

Typically, the objective is to observe both loses decreasing seeming, in order to avoid overfitting. The plot names correspond to the respective fold numbers; for instance, Figure A1 depicts the results for k = 1, Figure A2 for k = 2, and so forth.

## Appendix B

### Appendix B.1. Supplementary Data 2: ROC Curves

In Appendix B is presented the ROC curves across 25 epochs for each of the 10 folds. These plots illustrate the model’s classification performance across different epochs. Each ROC curve shows the trade-off between the True Positive Rate (Sensitivity) and False Positive Rate (1—Specificity) for various threshold settings. Specifically:

- The ROC curve of Bovine Milk (Class 0) is depicted as a blue solid line,
- The ROC curve of Goat Milk (Class 1) is represented as an orange solid line, and
- The ROC curve of Sheep Milk (Class 2) is illustrated as a green solid line.

The AUC value for each ROC curve is shown in the legend at the bottom right of the plot.

Ideally, the ROC curve should approach the upper left corner of the graph, indicating high classification performance compared to random guessing, which is represented by the diagonal dashed black line. The closer the curve is to this corner, the better the model is at distinguishing between classes.

The plot names correspond to the respective fold numbers; for instance, Figure A11 depicts the results for k = 1, Figure A12 for k = 2, and so forth
